# Reproducibility and stability of spirometer‐guided deep inspiration breath‐hold in left‐breast treatments using an optical surface monitoring system

**DOI:** 10.1002/acm2.13922

**Published:** 2023-02-27

**Authors:** Savino Cilla, Carmela Romano, Maurizio Craus, Pietro Viola, Gabriella Macchia, Mariangela Boccardi, Livia P. De Vivo, Milly Buwenge, Alessio G. Morganti, Francesco Deodato

**Affiliations:** ^1^ Medical Physics Unit Gemelli Molise Hospital Università Cattolica del Sacro Cuore Campobasso Italy; ^2^ Radiation Oncology Unit Gemelli Molise Hospital Università Cattolica del Sacro Cuore Campobasso Italy; ^3^ Radiation Oncology Department IRCCS Azienda Ospedaliero‐Universitaria di Bologna Bologna Italy; ^4^ DIMES, Alma Mater Studiorum Bologna University Bologna Italy

**Keywords:** ABC, breast, breath‐hold, SGRT, spirometer, surface‐guided, VMAT

## Abstract

The aim of this study was to evaluate the reproducibility and stability of left breast positioning during spirometer‐guided deep‐inspiration breath‐hold (DIBH) radiotherapy using an optical surface imaging system (AlignRT). The AlignRT optical tracking system was used to monitor five left‐sided breast cancer patients treated using the Active Breathing Coordinator spirometer with DIBH technique. Treatment plans were created using an automated hybrid‐VMAT technique on DIBH CTs. A prescribed dose of 60 Gy to the tumor bed and 50 Gy to the breast in 25 fractions was planned. During each treatment session, the antero‐posterior (VRT), superior‐inferior (LNG), and lateral (LAT) motion of patients was continuously recorded by AlignRT. The intra‐breath‐hold stability and the intra‐ and inter‐fraction reproducibility were analyzed for all breath‐holds and treatment fractions. The dosimetric impact of the residual motion during DIBH was evaluated from the isocenter shifts amplitudes obtained from the 50%, 90%, and 100% cumulative distribution functions of intra‐fractional reproducibility. The positional variations of 590 breath‐holds as measured by AlignRT were evaluated. The mean intra‐breath‐hold stability during DIBH was 1.0 ± 0.4 mm, 2.1 ± 1.9 mm, and 0.7 ± 0.5 mm in the VRT, LNG, and LAT directions, with a maximal value of 8.8 mm in LNG direction. Similarly, the mean intra‐breath‐hold reproducibility was 1.4 ± 0.8 mm, 1.7 ± 1.0 mm, and 0.8 ± 0.5 mm in the VRT, LNG, and LAT directions, with a maximal value of 4.1 mm in LNG direction. Inter‐fractional reproducibility showed better reliability, with difference in breathing levels in all fractions of 0.3 mm on average. Based on tolerance limits corresponding to the 90% cumulative distribution level, gating window widths of 1 mm, 2 mm, and 5 mm in the LAT, VRT, and LNG directions were considered an appropriate choice. In conclusion, despite the use of a dedicated spirometer at constant tidal volume, a non‐negligible variability of the breast surface position has been reported during breath‐holds. The real‐time monitoring of breast surface using surface‐guided optical technology is strongly recommended.

## 1 INTRODUCTION

Radiation‐induced cardiac and pulmonary complications represent a primary concern for radiotherapy of left‐sided breast cancer patients.^1^, ^2^, ^3^ Cardiovascular‐related mortality was found to be significantly higher after 15 yr of follow‐up than it is for patients with right‐sided breast cancer.^1^ In particular, Darby et al.^2^ reported that the rate of major coronary events increases linearly with the mean heart dose by 7.4% per Gy.

Deep‐inspiration breath‐hold (DIBH), by maximizing the distance between heart and breast, has proven to be a highly effective technique to reduce cardiac exposure during left‐breast radiotherapy.^4^, ^5^, ^6^ Furthermore, DIBH has proven to improve plan robustness, by reducing plan complexity without compromising plan deliverability.^7^ Different methods have been proposed to provide potential stable and reproducible repeated DIBHs during radiotherapy treatments. A common procedure is the use of a spirometer, able to suspend the patient's breath at a reproducible level with a DIBH technique. Several dosimetric studies have demonstrated potential advantages of ABC‐assisted left breast treatments in reducing the dose to cardiac organs‐at‐risk.^8^, ^9^, ^10^ Also, the reproducibility of the ABC system has been well studied in a few studies, all reporting average variability of the measured lung volumes below 2%.^11^

However, the inhaled (or exhaled) lung volume as measured by the ABC device is only a surrogate for the breast position, and potential benefits strongly depend on patient compliance and its ability to reproduce each breath‐hold. Therefore, unstable breath‐hold control could induce inaccurate target immobilization thereby resulting in potential sub‐optimal treatments. Some authors demonstrated that breast positional variations can occur in some patients even though the patient's tidal volume remains constant with ABC. Plathow et al.^12^ reported that for the same vital capacity, different breathing maneuvers (as abdominal and thoracic breathing) may lead to different chest wall expansion up to 1.9 cm. Remouchamps et al.^13^ also demonstrated that with an ABC device, overshoot can occur when the patient inhales more air than the pre‐determined fixed limit. More recently, using an optical surface‐tracking system Mittauer et al.^14^ reported large positional variations in some patients during DIBH due to their different breath hold techniques, causing increases in mean heart and coronary artery dose. Therefore, a real‐time control of individual breath‐hold stability and reproducibility is of great importance to ensure a safe and consistent radiation delivery in breast radiotherapy.

A more recent technology uses body surface photogrammetry by means of ceiling‐mounted surface scanning systems to monitor in real‐time the body surface position of the patient. This technology received an increasing acceptance and recognition as standard imaging technique for breast treatments, being continuously adopted in a large number of radiation oncology facilities.^15^ Advances in set‐up accuracy reported a reduction of positioning errors by around 40% on average when comparing laser alignment with surface imaging, leading to the possibility of tattoo‐free workflow and significantly reduced time for patient setup.^16^, ^17^, ^18^ Moreover, SGRT devices offer a unique possibility of evaluating “real‐time” intra‐DIBH stability and intra‐fractional reproducibility, potentially improving the safety and accuracy of dose delivery during each treatment fraction.^19^

The simultaneous integration of the ABC spirometer with an optical surface system has the potential to monitor and quantify the reproducibility and stability of each breath‐hold, and identify any variations resulting from different breathing maneuvers in a typical treatment fraction. To the best of our knowledge, the application of optical surface tracking in combination of ABC spirometer has been limited to only two studies. Wong et al.^20^ performed a study aiming to derive the benefits from using both spirometer and surface tracking in order to minimize treatment margin in lung radiotherapy. The efficacy of the technique in terms of tumor immobilization and tracking was proved to be reliable and to be potentially useful for dose escalation treatment. Mittauer et al.^14^ investigated the role of an in‐house optical tracking system in monitoring spirometer‐controlled BH for breast radiotherapy, reporting an improved reduction in cardiac toxicity.

The aim of this study was to evaluate the intra breath‐hold and the intra‐ and inter‐fraction reproducibility and stability of breast position using both ABC and SGRT systems. In particular, DIBH was applied using the ABC device in order to immobilize breast movement while the amount of residual breast motion during the consecutive breath‐holds was monitored based on the surface guided tracking device. Lastly, the dosimetric impact of residual movements during breath‐holds on breast coverage and lung and cardiac sparing was evaluated.

## 2 MATERIALS AND METHODS

### 2.1 Patient data, simulation, and volumes definition

Five consecutive left‐sided breast cancer patients, treated in DIBH using the Elekta ABC spirometer and the AlignRT (VisionRT, London, UK) surface scanning system, were included in this study. Patients underwent the CT‐scan simulation (Philips Big Bore) in the head‐first‐supine orientation with 2.0 mm slice thickness. Before final CT acquisition, a 2 days training is performed to explain procedure and coaching on the use of the ABC device. Patients underwent repeated DIBH to assess their ability to perform several breaths holds for at least 20 s at about 70%–80% of maximum deep inspiration. Patients positioning was obtained using a carbon fiber breast‐board (C‐Qual Breastboard, CIVCO Medical Solutions) with the ipsilateral arm placed above the head.

The clinical target volume (CTV) was defined to cover all breast tissues. The planning target volume (PTV) was defined as the CTV plus a 5 mm margin. The boost clinical target volume (CTV‐boost) consisted of the tumor bed, as marked by the surgery metal clips, postoperative serum swelling and surgical scars. The PTV‐boost was created by adding an isotropic 7 mm margin to the CTV‐boost. The lung, heart, and contralateral breast were identified as organs‐at‐risk (OARs).

### 2.2 Breath‐hold guidance and monitoring: ABC and AlignRT devices

The ABC device consists of a mouthpiece connected to a spirometer (to measure air‐flow) and coupled to a balloon valve.^10^ A nose clip ensure that patients breathe through the mouthpiece connected to the device. The valve is closed at approximatively 75% of the maximum inhalation volume, as determined during training, allowing a comfortable level of breath‐hold.

**FIGURE 1:**
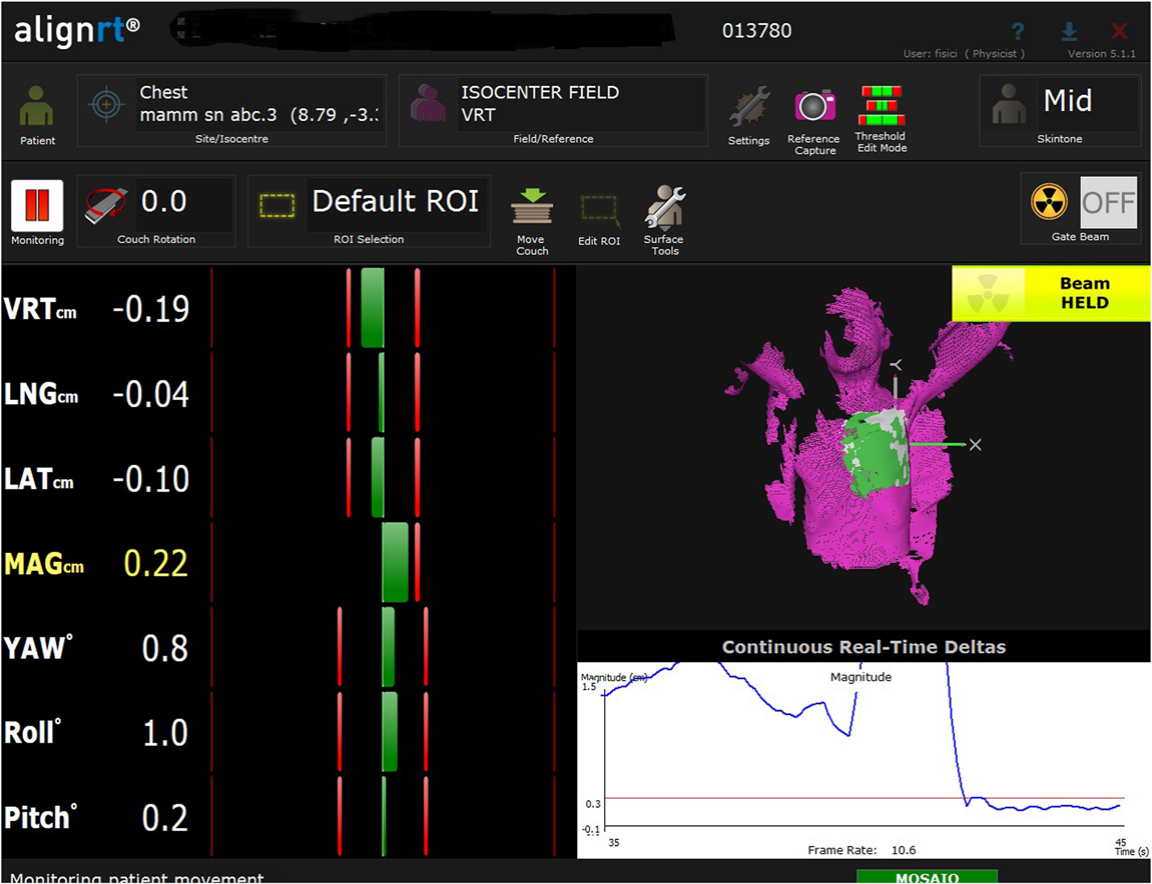
Screenshot of the AlignRT software control showing the region of interest for DIBH monitoring of a breast target for a representative patient.

The AlignRT infrared optical tracking system was used to continuously monitor the interfraction, intrafraction, and intrabreath‐hold motion in the anterior‐posterior (VRT), lateral (LAT), and superior‐inferior (LNG) directions during the DIBHs. The accuracy of the AlignRT system has been shown to be submillimeter,^21^ and the calibration was checked daily. This system was installed in the treatment room with a three‐pod configuration. Before the start of acquisition, a Region‐Of‐Interest (ROI) on the surface body need to be defined by the user in order to monitor the patient's position. The deviations of the surface position are calculated between this user‐defined ROI and the reference surface and are recorded in a file text with frame rate of 5 Hz, the so‐called RealTimeDelta tool, for post‐processing. The reference image can be obtained directly or by importing the body surface from the planning CT. The ROI was standardized for every patient in order to include the left breast with a 5 cm isotropic margin. In this study, the AlignRT system was not used for setup positioning purposes but only to monitor the patient during the treatment fractions because our main goal was the assessment of DIBH stability and reproducibility. Figure 1 reports a screenshot of the AlignRT software.

### 2.3 Treatment planning

Treatment planning was performed on CT‐scan obtained in DIBH, using a commercial treatment planning system (Pinnacle,^3^ Philips Radiation Oncology Systems, Fitchburg, WI). Patients were treated with an automated hybrid‐VMAT (HVMAT) technique and simultaneous integrated boost to surgical cavity. Dose prescription was 50 and 60 Gy in 25 fractions to the breast and to the surgical cavity, respectively. The HVMAT approach delivered 75% of prescription dose to the entire breast through tangential beams and 25% through two partial VMAT arcs. Planning details are reported in a previous paper.^22^ The dosimetric goals for both PTVs were as follow: coverage of the PTV by the 95% isodose (V95% ≥ 95%), near‐minimal dose greater than 90% of prescription dose (D98% ≥ 90%) and near‐maximal dose not exceeding 107% of the prescribed dose (D2% ≤ 107%). For the heart, the main objective was to keep the mean dose below 4 Gy.^23^ The dose‐volume objectives for the ipsilateral lung were defined according to recent guidelines for radiation‐induced lung toxicity as follows: V5 < 50%, V10 < 35%, V20 < 20%.^24^ The mean dose and D1cc (dose to 1cc breast volume) to the contralateral breast was restricted to less than 2 and 5 Gy, respectively. Dose calculation was performed with the collapsed cone convolution algorithm with a 2 mm dose grid resolution.

### 2.4 Setup verification

At the treatment machine, the patient positioning was initially performed using skin marks, then shifting the patient to the treatment isocenter. Next, a fast 200° gantry kV cone beam CT (kV‐CBCT) was acquired in breath‐hold condition; setup corrections were applied using an automatic 6‐DOF registration to the DIBH planning CT with the XVI software (Elekta) and the Hexapod treatment couch. Then, the AlignRT system was used to capture the DIBH body surface directly after the CBCT position correction for intrafraction treatment monitoring. After the patient positioning, the treatment was delivered in DIBH by a VersaHD linear accelerator (Versa HD, Elekta, Stockholm, Sweden) using a hybrid‐VMAT technique. Due to inter‐fractional setup variations, a new benchmark skin surface image was established at the beginning of each treatment session. This way, the breast surface is linked to the anatomy as visualized by CBCT by taking the SGRT image capture at the end of CBCT acquisition and registration. This procedure should allow a near perfect registration with the SGRT‐based breast surface and CBCT‐based internal anatomy.

### 2.5 Data analysis

The real time data text files were extracted from AlignRT software and imported and analyzed in Excel (v2010, Microsoft). Mean, median and standard deviations (SD) values together with 95% confidence intervals (CI95%) were used to quantify the metrics stability and reproducibility. Correlations between spirometer and optical surface imaging deviations were investigated.

The reproducibility and stability of breast external surface position during DIBHs was evaluated by means of the following metrics, as described by Cervino et al.^25^ and Mittauer et al.^14^

**FIGURE 2:**
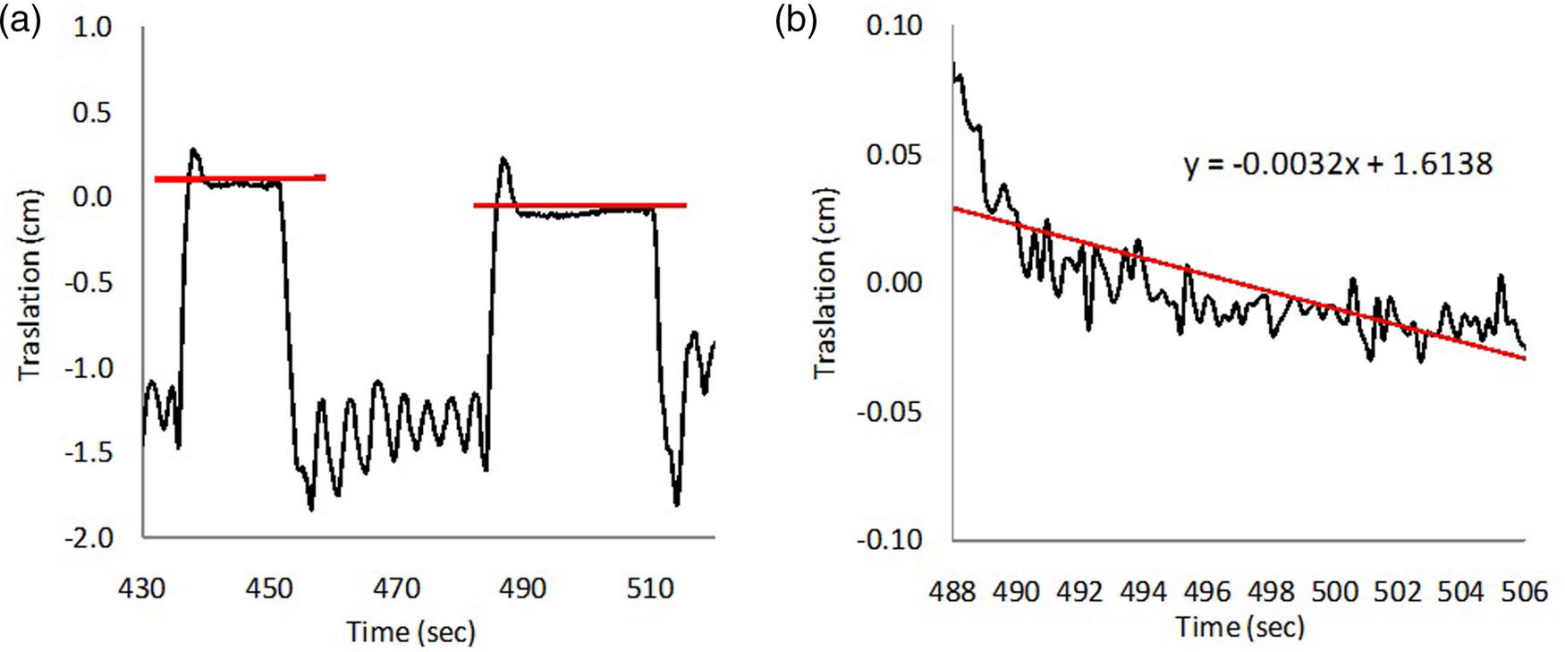
Graphical representations of (a) reproducibility and (b) stability parameters. Black line represents the deviation in mm over time during one DIBH cycle and red line represents the linear fit plot of the black signal.

For each breath‐hold sequence during a treatment fraction, the intra‐fraction *reproducibility* R was defined as the maximum difference between different DIBH levels:

$$R=\max_{i=\left[1,n\right]}\left\{d_{i}\right\}-\min_{i=\left[1,n\right]}\left\{d_{i}\right\}$$

where *n* and *d*_i_ are the number of DIBHs and the average level of each DIBH in the sequence, as shown in Figure 2a. Large *R* values represents large varied DIBH levels and then poor reproducibility.

The inter‐fraction *reproducibility* was determined as the difference between the mean breath‐hold level per fraction and the mean breath‐hold level during the first fraction.

The *stability S* was defined as the linear amplitude deviation during a DIBH. The gradient of the regression fitted line was multiplied with the DIBH‐time interval providing an amplitude deviation during a DIBH:

$$S=\max_{i=\left[1,n\right]}\left\{m_{i}\cdot \Delta t\right\}$$

where *m* is the slope of the linear fit line to each DIBH and Δ*t* is the DIBH time range (usually 20–30 s), as shown in Figure 2b. Using these definitions. reproducibility and stability are expressed in mm, useful in order to be compared with patient setup units.

### 2.6 Dosimetric impact of residual motion

An evaluation of the dosimetric impact of residual motion during breath‐hold on target coverage and OARs irradiation was performed following the suggestions of Kugele et al.^32^ In details, following the aforementioned definition, we calculated the stability reproducibility in the VRT, LAT, and LNG directions corresponding to the 50%, 90%, and maximal (100%) cumulative distribution functions for the deviations. For example, the 90% cumulative distribution function for the deviations (in millimeters) along one direction means that for 90% of DIBHs, the stability reproducibility is less or equal than X mm in that direction. Therefore, for each shift corresponding to the 50%, 90%, and 100% values of cumulative distributions, the dose was recalculated using the original plan. For each plan, the breast coverage in terms of D98% and the irradiation of ipsilateral lung and the heart in terms of mean dose, D2% and V20Gy were obtained from the corresponding DVHs.

## 3 RESULTS

Each treatment fraction was completed using 4–6 breath‐holds. A total of 118 out of 125 fractions had surface imaging data available for analysis, corresponding to 590 analyzed breath‐holds data. Breath‐holds during image guidance (portal imaging, CBCT) were excluded from this analysis.

**FIGURE 3:**
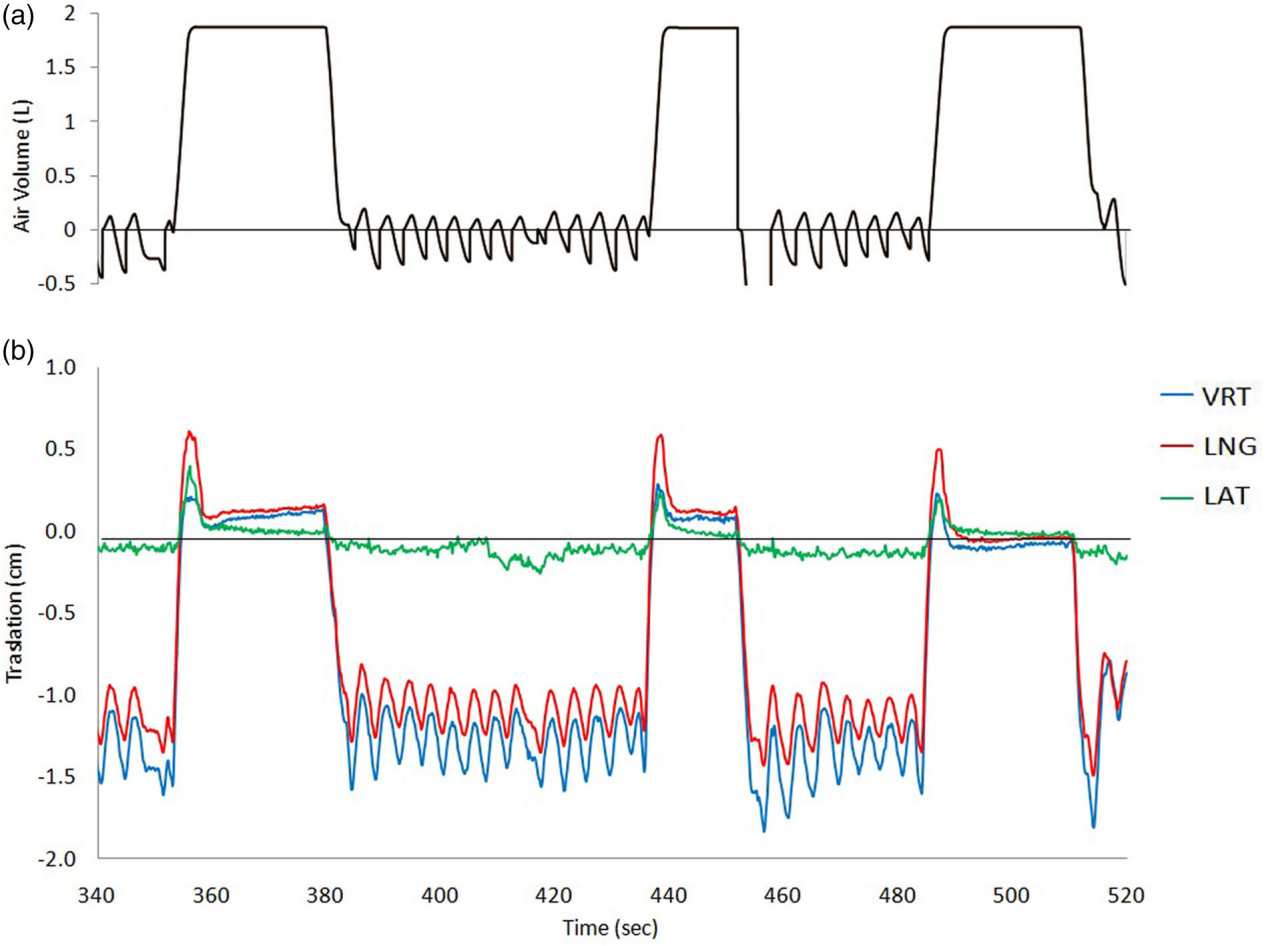
(a) The spirometer signal is shown in volume units (L). (b) The continuous signals of patient monitoring by the surface‐guided system for the vertical (VRT, anterior‐posterior in blue), longitudinal (LNG, superior‐inferior in red), and lateral (LAT, in green) coordinates. In particular, this patient displays some breast relaxation during intra breath‐hold time, most in the LNG direction, not reflected in the perfectly flat spirometer signal.

Figure 3 shows an example of the ABC spirometer (upper) and AlignRT time trajectories along the three spatial directions (lower) for three breath‐holds for a representative patient.

**FIGURE 4:**
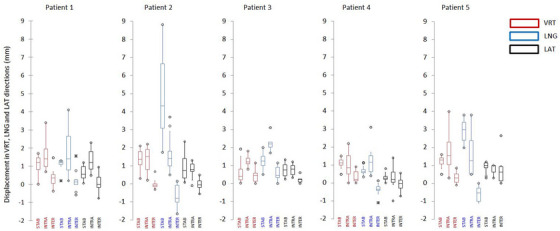
Box plots of stability and intra‐ and inter‐fraction reproducibility in the VRT (red), LNG (blue), and LAT (black) directions during all breath‐holds for the five evaluated patients (STAB = Stability, INTRA = intra‐fraction reproducibility, and INTER = inter‐fraction reproducibility).

The stability, inter‐ and intrafraction variability of the VERT, LNG, and LAT amplitudes for all patients is shown in Figure 4 as box‐plots and reported in Table 1.

**TABLE 1:** Stability, intra‐ and inter‐fraction variability of the VRT, LNG, and LAT amplitudes for all patients

	Intra‐breath‐hold			Intra‐fraction			Inter‐fraction		
	Stability			Reproducibility			Reproducibility		
	VRT (mm)	LNG (mm)	LAT (mm)	VRT (mm)	LNG (mm)	LAT (mm)	VRT (mm)	LNG (mm)	LAT (mm)
Patient 1									
mean ± SD	1.0 ± 0.4	1.3 ± 0.2	0.6 ± 0.3	1.6 ± 1.0	1.9 ± 1.4	1.3 ± 0.7	0.3 ± 0.6	0.1 ± 0.6	0.1 ± 0.5
*Maximum*	1.7	1.3	1.2	3.4	4.1	2.3	1.5	1.6	1.0
Patient 2									
mean ± SD	1.3 ± 0.5	4.8 ± 2.0	0.9 ± 0.7	1.3 ± 0.6	1.7 ± 1.0	0.8 ± 0.4	0.0 ± 0.2	−0.7 ± 0.5	0.0 ± 0.3
*Maximum*	2.1	8.8	2.3	2.2	3.7	1.3	0.7	0.1	0.5
Patient 3									
mean ± SD	0.6 ± 0.5	1.1 ± 0.4	0.7 ± 0.3	1.3 ± 0.4	2.3 ± 0.5	0.8 ± 0.4	0.5 ± 0.4	0.6 ± 0.5	0.2 ± 0.2
*Maximum*	1.9	2.0	1.3	1.8	3.1	1.2	1.1	1.1	0.6
Patient 4									
mean ± SD	1.2 ± 0.2	2.7 ± 0.5	0.9 ± 0.2	1.8 ± 1.3	1.6 ± 1.4	0.8 ± 0.3	0.3 ± 0.4	1.0 ± 1.3	0.8 ± 1.0
*Maximum*	1.6	3.8	1.2	4.0	3.8	1.1	0.9	1.1	2.7
Patient 5									
mean ± SD	1.0 ± 0.2	0.7 ± 0.2	0.3 ± 0.2	1.0 ± 0.8	1.2 ± 0.8	0.3 ± 0.7	0.3 ± 0.3	−0.4 ± 0.3	−0.1 ± 0.4
*Maximum*	1.5	1.1	0.8	2.2	3.1	1.4	0.9	0.1	0.6
All									
mean ± SD	1.0 ± 0.4	2.1 ± 1.9	0.7 ± 0.5	1.4 ± 0.8	1.7 ± 1.0	0.8 ± 0.5	0.3 ± 0.4	0.3 ± 0.6	0.2 ± 0.5
*Maximum*	2.1	8.8	2.3	4.0	4.1	2.3	1.5	1.6	2.7

Table 2 compares the mean intra‐DIBH stabilities and intra‐fraction surface reproducibility obtained in our study with those evaluated by Fassi et al.^26^ using spirometric‐DIBH and Xiao et al.^27^ using voluntary DIBH. The percentage of DIBH lying within a specified magnitude is also reported. Our data report superior intra‐fraction reproducibility but worse results in terms of intra‐DIBH stability with respect to voluntary DIBH. Thresholds of at least 2, 3, and 4 mm in the LAT, VRT, and LNG directions are indicated in order to capture the variability in DIBH over a full treatment fraction (i.e., intrafraction) for 95% of DIBHs.

**TABLE 2:** Intra‐DIBH stabilities and intra‐fraction surface reproducibility obtained in our study with those evaluated by Fassi et al.^28^ using spirometric‐DIBH and Xiao et al.^29^ using voluntary DIBH

	Intra‐breath‐hold			Intra‐fraction		
	Stability			Reproducibility		
	VRT (mm)	LNG (mm)	LAT (mm)	VRT (mm)	LNG (mm)	LAT (mm)
This study	1.0	2.1	0.7	1.4	1.7	0.8
% within 2 mm	98.6	60.1	97.1	80.0	67.3	96.4
% within 3 mm	100	78.3	100	96.4	81.8	100
% within 4 mm	100	87.7	100	98.2	98.2	100
% within 5 mm	100	89.9	100	100	100	100
Fassi et al.^28^	1.4	1.8	0.7	2.2	2.3	1.9
Xiao et al.^29^	0.7	0.6	0.5	2.2	2.0	1.5

The percentage of DIBHs lying within a specified magnitude is reported

### 3.1 Stability, intra‐ and inter‐fraction reproducibility analysis

The overall mean stability of the breath‐hold levels during DIBH was 1.4 ± 0.6 mm, 2.6 ± 0.9 mm, and 0.8 ± 0.4 mm in the VRT, LNG and LAT directions, respectively. Overall intra‐ and inter‐fractional reproducibility of amplitudes were found 1.4 ± 0.8 mm, 1.7 ± 1.0 mm, and 0.8 ± 0.5 mm, and 0.3 ± 0.4 mm, 0.3 ± 0.6 mm, and 0.2 ± 0.5 mm in the VRT, LNG, and LAT directions, respectively. Intra breath‐hold displacements up to 9 mm was observed for one patient in the LNG direction. The intra breath‐hold variability was negligible (<1 mm) only in the LAT direction. With respect to intra‐fraction reproducibility, displacement up to 4 mm between different breath‐holds in the same session were observed in the VRT and LNG directions, despite the almost constant inspired volume supplied by the ABC spirometer.

**FIGURE 5:**
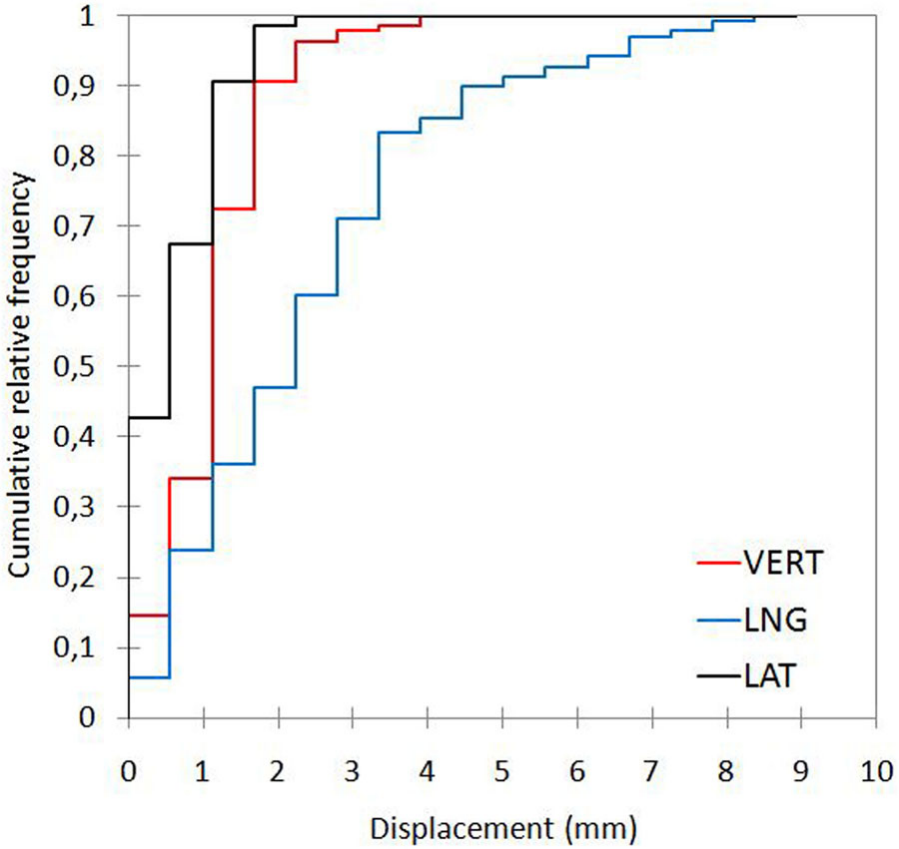
Cumulative distribution functions for deviations in millimeters along the three directions for the stability metric.

Figure 5 shows the cumulative distribution functions for deviations in millimeters along the three directions for the stability metric. The 50%, 90%, and 100% cumulative probabilities provided reproducibility values of 0.6, 1.1, and 2.2 mm in the LAT direction, 1.1, 1.7, and 3.9 mm in the VRT direction, and 2.2, 5.0, and 8.9 mm in the LNG direction, respectively.

The percentage of breath‐holds with a change of stability amplitude less than 3 mm was 100% in the VRT and LAT directions, but 78% in the LNG direction.

**FIGURE 6:**
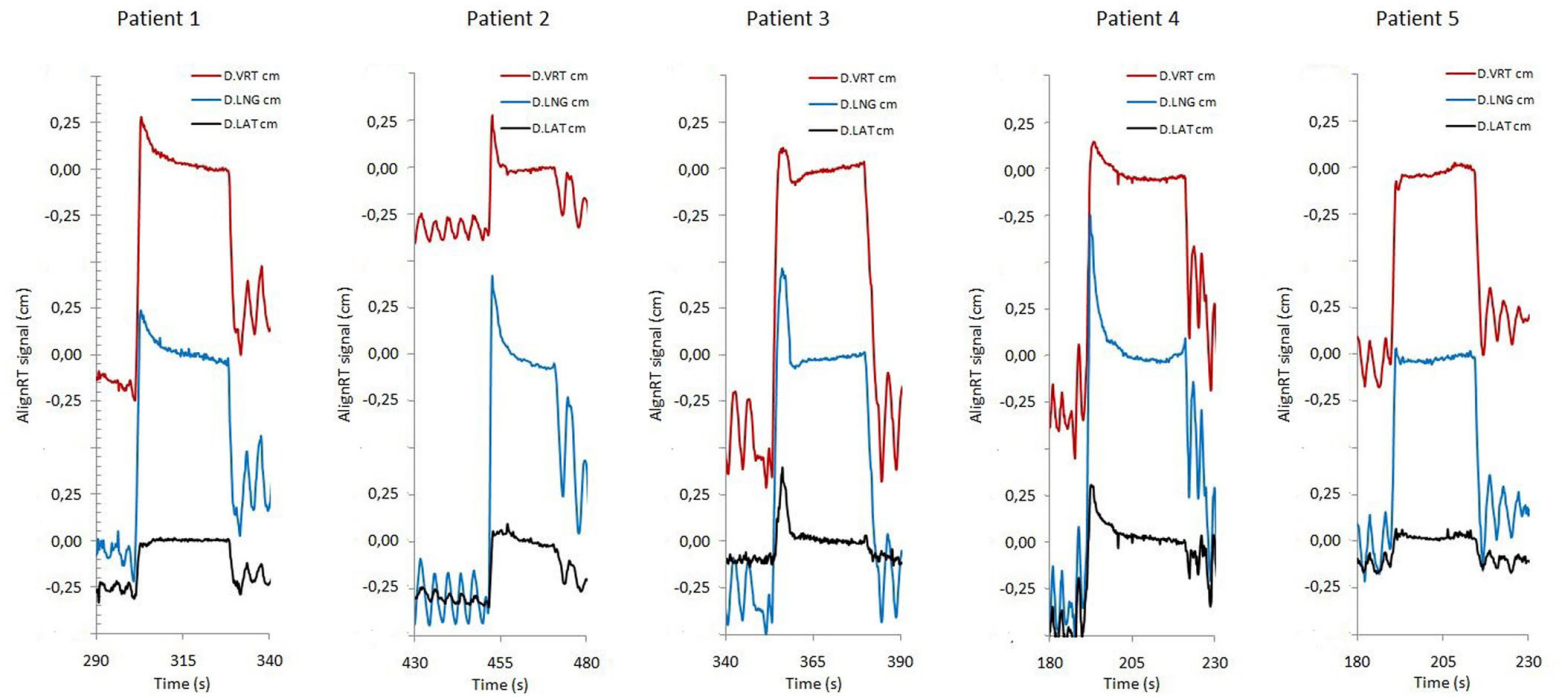
Differences between breathing maneuvers of all patients along the three spatial directions.

In order to highlight the differences in breathing maneuvers, Figure 6 shows the different trajectories along the three spatial directions for all patients.

For each patient, a Friedman's test followed by a Nemenyi post‐hoc analysis for coupled breathing amplitudes was performed for all breath‐holds during the treatment. Although the inter‐fraction reproducibility during the whole treatment session was found quite small for each patient, the resulting test significance (*p* < 0.05) suggested that the breathing amplitudes between individual DIBH maneuvers were significantly different. This was obtained for all patients.

No significant correlations were found between the ABC breath‐hold levels and the surface monitoring deviations for the VRT (*R* ^2^ = 0.10), LNG (*R* ^2^ = 0.20), and LAT axes (*R* ^2^ = 0.14).

### 3.2 Dosimetric impact of residual motion

**FIGURE 7:**
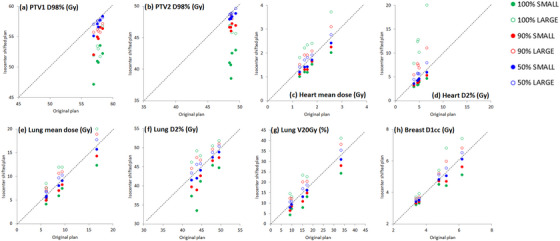
Plots of the main dosimetric metrics for PTV, heart and lung of the isocenter‐shifted plans versus the original plans. The dashed lines correspond to the bisector, that is, the locus of points where the dosimetric metrics have the same values for both plans. Each point represents an individual patient. Blue, red, and green colors correspond to the 50%, 90%, and 100% cumulative distribution levels.

The evaluations for the dosimetric impact of stability reproducibility are presented in Table 3 and Figure 7.

**TABLE 3:** Changes in dosimetric metrics due to intrafractional isocenter reproducibility during DIBH obtained for the three cumulative distribution levels at 50%, 90%, and 100%

		Isocenter shifted plans					
		50%		90%		100%	
	Original plan	LARGE	SMALL	LARGE	SMALL	LARGE	SMALL
*PTV1*							
D98% (%)	57.6 (57.0–58.4)	57.6 (57.0–58.1)	57.0 (55.1–58.3)	56.2 (55.7–57.1)	54.9 (52.0–56.5)	53.4 (51.7–56.7)	50.9 (47.1–53.5)
*PTV2*							
D98% (%)	48.8 (48.4–49.4)	48.9 (48.6–49.6)	48.2 (47.9–48.8)	48.3 (48.0–48.8)	46.7 (46.0–47.2)	46.6 (45.7–48.0)	41.1 (38.5–43.0)
*Heart*							
Dmean (Gy)	1.7 (1.3–2.6)	1.9 (1.4–2.8)	1.6 (1.2–2.4)	2.1 (1.5–3.1)	1.5 (1.1–2.2)	2.5 (1.8–3.7)	1.4 (1.0–2.0)
D2% (Gy)	5.0 (3.9–6.6)	5.8 (4.4–7.9)	4.5 (3.5–5.9)	7.7 (5.4–11.0)	4.1 (3.2–5.3)	12.6 (7.8–20.1)	3.6 (2.9–4.7)
*Lung*							
Dmean (Gy)	9.5 (6.1–16.7)	10.2 (6.6–17.7)	8.8 (5.5–15.7)	11.0 (7.0–18.9)	7.9 (4.9–14.2)	12.0 (7.5–20.1)	6.9 (4.1–12.3)
D2% (Gy)	45.7 (42.5–49.6)	46.7 (43.3–50.3)	44.7 (41.4–48.8)	47.8 (44.5–51.0)	43.0 (38.9–47.3)	49.0 (46.1–51.7)	40.3 (33.4–45.3)
V20Gy (%)	17.1 (9.3–33.5)	18.4 (10.5–35.3)	15.4 (7.8–30.8)	20.5 (12.0–38.0)	13.6 (6.3–27.9)	22.8 (12.9–41.0)	11.3 (4.2–24.3)
*Contralateral breast*							
Dmean (Gy)	4.7 (3.4–6.2)	4.8 (3.5–6.5)	4.6 (3.4–6.1)	5.1 (3.6–6.8)	4.3 (3.3–5.6)	5.4 (3.7–7.4)	4.1 (3.2–5.1)

Table 3 reports the changes of the main dosimetric metrics for the PTVs coverage and heart and lung irradiation due to the intra‐fractional isocenter reproducibility during DIBH obtained for the three cumulative distribution levels at 50%, 90%, and 100% for all patients. Following the suggestions of the study of Kugele et al.,^32^ the shifts were categorized in two main groups, namely shifts producing the largest (LARGE) and the smallest (SMALL) impact on dose distribution for OARs.

As expected, the D98% metric for the coverage of the two target volumes was always smaller for all the isocenter‐shifted plans with respect to original plans, since any deviations from original isocenter correspond to a shift of PTVs outside the tailored treatment field of VMAT segments. In particular, for the PTV1 and PTV2, median values of D98% decreased from 57.6 Gy and 48.8 Gy of original plans to minimal values of 57.0, 54.9, and 50.9 Gy, and 48.2, 46.7, and 41.1 Gy for shifts corresponding to 50%, 90% and 100% values of cumulative distribution functions.

For the heart and the ipsilateral lung, the worse deterioration of all dosimetric metrics is observed when the isocenter shifts are obtained in the right LAT, posterior VRT and caudal LNG directions, because these shifts correspond to a greater exposure of these organs in the treatment field. In this case, the heart mean doses increased from 1.7 Gy in the original plan up to 1.9, 2.1, and 2.5 Gy. Similarly, isocenter shifts in the opposite directions contributes to a removal of lung and heart from the treatment fields and the corresponding dose metrics will significantly improve, as reported in Table 3.

Figure 7 shows the plots of the main dosimetric metrics for PTV, heart and lung of the isocenter‐shifted plans versus the original plans. The dashed lines correspond to the bisector, that is, the locus of points where the dosimetric metrics have the same values for both plans. Each point represents an individual patient. Blue, red and green colors correspond to the 50%, 90%, and 100% cumulative distribution levels.

## 4 DISCUSSION

The benefits of spirometer‐guided DIBH have been extensively reported in literature. According to Bergom et al.’s review article,^26^ this strategy demonstrated the possibility to decrease the mean heart dose by 25%–67% and mean LAD dose by 20%–73%, respectively compared with the same patients planned with free breathing. Also, the reproducibility of the volume inhaled/exhaled by the patient measured by the ABC device was found very high. Kaza et al.^11^ evaluated the intra‐session reproducibility of ABC‐induced lung volumes was evaluated and compared with that reached by applying the clinical standard of operator‐guided self‐sustained breath‐holds on healthy volunteers. The average ABC variation of measured lung volumes was 1.8%, compared to the 4.1% variability observed on average with self‐sustained breath‐holding.

However, this high lung volume reproducibility hides the implicit assumption that consistent lung volumes provide consistent positioning, that is, a low variability in breast/thorax positions for all patients. A few studies have questioned the lung volume as a perfect surrogate for anatomical position. For example, Plathow et al.^12^ demonstrated that the position of the lung and chest wall were strongly dependent on the type of breathing movement, namely “abdominal breathing,” “thoracic breathing,” and “normal breathing” as measured using dynamic magnetic resonance imaging and fiducial markers.

In the present study, the intra‐breath‐hold stability and intra and inter‐fraction reproducibility of spirometer‐guided DIBH were quantitatively evaluated on five left‐breast cancer patients. This aim was achieved monitoring all individual breath‐holds in ABC‐guided treatments using the AlignRT surface‐guided optical system. Although the average breath‐hold variability was small for both stability and reproducibility, outliers are not uncommon. The intra‐breath‐hold stability was found on average 1.0, 2.1, and 0.7 mm in the VRT, LNG, and LAT directions. One patient reported a mean value of 4.8 mm in the LNG direction, with maximal displacement up to 8.9 mm. This is a quite unexpected result but it highlighted the suggestion that particular breathing maneuvers or chest and abdominal relaxation may translate in large displacements of breast surface during DIBH despite the constant lung volume, as reported in Figure 5. This supports our conclusion that volume invariance during single or repeated DIBHs is not a reliable indicator of a patient's thoraco–abdominal surface's geometric stability and reproducibility.

The intra‐fraction reproducibility in the VRT, LNG, and LAT directions was reported in Table 2, providing better or comparable results with respect to those reported by others authors.^27^, ^28^, ^29^, ^30^ Fassi et al.^27^ used an infrared optical tracking to record the coordinates of seven passive markers placed on the patient's thoraco–abdominal surface during DIBHs. The results showed displacements of the external surface between different sessions up to 6.3 mm along a single direction, even at constant inspired volumes. The median value of the inter‐fraction variability in the position of breast passive markers was 2.9 mm (range: 1.9–4.8 mm), 3.6 mm (range: 2.2–4.6 mm), and 4.3 mm (range: 2.8–6.2 mm) in the LAT, VRT, and LNG, respectively. Similarly, Xiao et al.^28^ evaluated the intra‐DIBH stability and the intrafraction reproducibility during voluntary DIBH of 58 patients using the AlignRT real‐time surface imaging. The authors reported values ≤0.7 mm and ≤2.2 mm for stability and intrafraction variability, respectively, and adopted a 7 mm threshold for the 3D magnitude in order to account for 95% of total intrafraction variability.

Surface imaging has seen a significant growth in application for DIBH in recent years, offering various benefits as real‐time motion management, improved setup accuracy, and faster setup times.^31^ In particular, the real‐time monitoring of the patient's surface avoids the issue that variations in inhalation volume or breathing type could cause a measurable difference in the positioning of the target volume. Furthermore, because of the increasing widespread use of VMAT and/or simultaneous integrated boost strategy, the importance of optimal positioning of the breast surface is becoming more important for accurate dose deposition. Several investigations have found that changes of about 5 mm in breast surface could have a strong impact on cardiac toxicity or breast underdosage.^32^, ^33^ For example, Kugele et al.^32^ investigated the impact of intra‐fractional motion during DIBH on the heart, left anterior descending coronary artery and ipsilateral lung for left‐sided breast cancer. Although the intra‐fractional reproducibility was within 1 mm for most of the treatment sessions, several sessions reported intra‐fractional DIBH isocenter reproducibility up to 5 mm, resulting in large dosimetric effects on the target volume and organs at risk. In particular, near‐maximal doses for heart and LAD were increased up to 2 and 5 Gy, respectively.

In this paper the dosimetric impact of residual motion during DIBH was evaluated following the suggestions of Kugele et al.^32^ For isocenter shifts related to the 50%, 90%, and 100% cumulative distributions, we observed a progressive worsening of targets coverage in terms of D98% from 1% to about 15%. On the contrary, the dosimetric impact on heart was found less prominent, with a maximal increase of mean dose of only 0.8 Gy also for the large isocenter shifts related to the 100% cumulative distribution. This is because the use of DIBH technique was able to move the heart well out the treatment field during simulation CT for all patients, and the applied isocenter shifts are not sufficient to move significantly the heart within the irradiation fields. As expected, the dosimetric impact is more pronounced for D2%, representing the near‐maximal dose, since isocenter shifts may expose very small volumes of heart to high isodoses, thus increasing high point doses.

It must be underlined that the use of cumulative probability distributions for evaluating the dosimetric impact of the residual motion during DIBH may greatly help to introduce tolerance limits for the isocenter deviation in every individual DIBH. Assuming tolerance limits corresponding to the 90% cumulative distribution level, our results reported that corresponding residual motion translated in a median D98% reduction less than 5% and in a median increase of heart and lung doses of 0.4 Gy and 1.5 Gy with respect to the original plan, respectively. This means that gating window widths of 1 mm, 2 mm, and 5 mm in the LAT, VRT, and LNG directions may be considered an appropriate choice.

Recently, a study performed by Gnerucci et al.^34^ aimed to prove the reliability of selected beam‐hold thresholds and their impact on intra‐fractional motion management during the DIBH treatment of left breast cancer patients. The authors selected a threshold for the SGRT shift equal to 5 mm and reported a median value for mean and maximum values of SGRT shifts equal to 2.4 mm (95% CI: 1.7–3.1 mm) and 4.1 mm (95% CI: 3.3–5.6 mm), respectively. The amplitude of the SGRT shift was found higher than 3 and 4 mm for 30% and 10% of treatment time, respectively.

Another finding in the present study is the lack of correlation between breast surface positions and inspired tidal volumes, suggesting that the surface displacements do not depend on the variability of the (almost constant) tidal lung volume. For example, patient 2 exhibited a mean surface displacement of about 5 mm in the LNG direction but with a variability of the mean inspired volume of only 0.04 L (i.e. ≤ 2%). As shown in Figure 5, even during a single DIBH, a constant inspired volume can be associated with surface displacements up to several mm due to thoracic and abdominal relaxation.

A few limitations of this study should be highlighted. A first limitation of our study could be the small number of patients. Nevertheless, in our opinion, this investigation is valuable as an exploratory study, and the almost 600 analyzed breath hold cycles are sufficient for its purposes. A second limitation is that the dosimetric impact of residual motion during DIBH was evaluated assuming rigid isocenter translations, that is, the whole patient moves to the new isocenter coordinates. This assumption is not completely true because the geometrical relationship between the heart and the breast is not constant, and the use of a deformable patient model will supply a better accuracy in order to account for breath‐hold variations. However, because the rigid translations were of few millimeters, our evaluation should provide a reasonable approximation of the dosimetric impact on breast, heart and lung volumes. Another limitation is represented by the choice to perturb the plans without considering the real movement of each single patient, but applying the same shift vectors for the whole patient group. A more “personalized” approach could be adopted to evaluate the dosimetric impact of effective residual motion during DIBH.

Based on the present findings, we concluded that the patient's ability to reproduce consistent DIBH maneuvers is crucial for a reliable use of ABC system. Particularly, the high reproducibility of ABC lung tidal may provide a false sense of security that the patient's breast is accurately positioned within the treatment field. Therefore, a verification tool for spirometer‐guided DIBH treatments is strongly recommended. Real time surface‐based optical systems are a viable option. The main expected advantage is represented by the automated gating mechanism, able to automatically stop the irradiation if the breath‐hold levels fall out of the gating window. Based on tolerance limits corresponding to the 90% cumulative distribution level, gating window widths of 1 mm, 2 mm, and 5 mm in the LAT, VRT, and LNG directions are considered an appropriate choice.

## 5 CONCLUSION

Even though the use of a dedicated spirometer at constant tidal volume, a non‐negligible variability of the breast surface position has been reported during breath‐holds. The real‐time monitoring of breast surface using surface‐guided optical technology allows the detection of even small breath‐hold pattern deviations, ensuring treatment stability, and reproducibility for every individual patient.

## AUTHOR CONTRIBUTIONS

Savino Cilla, Carmela Romano, Gabriella Macchia, Milly Buwenge, Alessio G. Morganti, and Francesco Deodato were involved in study design and data interpretation. Maurizio Craus, Pietro Viola, Mariangela Boccardi, and Livia P. De Vivo were involved in the data acquisition and analysis. Savino Cilla conducted statistical analyses. Savino Cilla drafted the original manuscript. All authors critically revised the manuscript and approved the submitted version.

## FUNDING

The authors received no specific financial support for the research, authorship, and/or publication of this article.

## CONFLICT OF INTEREST

All authors declare no competing interests.
